# Targeted pruning of a neuron’s dendritic tree via femtosecond laser dendrotomy

**DOI:** 10.1038/srep19078

**Published:** 2016-01-07

**Authors:** Mary Ann Go, Julian Min Chiang Choy, Alexandru Serban Colibaba, Stephen Redman, Hans-A. Bachor, Christian Stricker, Vincent Ricardo Daria

**Affiliations:** 1 Eccles Institute of Neuroscience, John Curtin School of Medical Research, The Australian National University, Canberra, ACT, Australia; 2 Department of Quantum Science, Research School of Physics and Engineering, The Australian National University, Canberra, ACT, Australia; 3 Medical School, College of Medicine, Biology and Environment, The Australian National University, Canberra, ACT, Australia

## Abstract

Neurons are classified according to action potential firing in response to current injection. While such firing patterns are shaped by the composition and distribution of ion channels, modelling studies suggest that the geometry of dendritic branches also influences temporal firing patterns. Verifying this link is crucial to understanding how neurons transform their inputs to output but has so far been technically challenging. Here, we investigate branching-dependent firing by pruning the dendritic tree of pyramidal neurons. We use a focused ultrafast laser to achieve highly localized and minimally invasive cutting of dendrites, thus keeping the rest of the dendritic tree intact and the neuron functional. We verify successful dendrotomy via two-photon uncaging of neurotransmitters before and after dendrotomy at sites around the cut region and via biocytin staining. Our results show that significantly altering the dendritic arborisation, such as by severing the apical trunk, enhances excitability in layer V cortical pyramidal neurons as predicted by simulations. This method may be applied to the analysis of specific relationships between dendritic structure and neuronal function. The capacity to dynamically manipulate dendritic topology or isolate inputs from various dendritic domains can provide a fresh perspective on the roles they play in shaping neuronal output.

Studying the input-output transfer function of neurons is essential to understanding information processing in the brain. Neurons can be classified by electrophysiological means via their distinctive temporal firing patterns of action potentials following the injection of current at the soma^1^,^2^,^3^,^4^. A neuron’s firing pattern is shaped by the composition and distribution of ion channels in the membrane and by dendritic morphology^5^,^6^,^7^.

Neurons have a functional polarity; that is, they have a uni-directional flow of information. Inputs from other cells arrive at the dendrites while the output from their integration is sent to other neurons through the axon via synapses. Synaptic integration is highly dependent on the timing and location of inputs, the properties of dendrites and the interaction between cellular compartments. Inputs to various dendritic domains, e.g. apical or basal, are hypothesized to play specific roles in generating an action potential. Yet the functional significance of inputs to these dendritic domains remains cryptic^8^,^9^,^10^. To systematically study their functions, the morphological complexity of dendritic structure can be modified by isolating inputs from different domains by physically cutting dendrites (dendrotomy).

Several mechanical techniques^11^,^12^,^13^,^14^,^15^ have been used to disconnect neurons from their processes in brain slices. These techniques, however, are invasive and incur collateral damage to the cell and the surrounding tissue. As such, their application has been mostly limited to dissection of the main apical dendrite. The challenge is to realize a tool and establish an efficient opto-electrophysiological protocol that can access dendrites embedded deep within brain tissue and perform highly targeted dendrotomy while keeping the neuron functional and the surrounding neuropile largely intact.

Non-linear multi-photon processes can facilitate highly localized dissection of dendrites within brain tissue. The key is to use a highly penetrating near-infrared (NIR) femtosecond (fs) pulse laser focused to a diffraction-limited focal volume. Cutting is achieved at the focus where a cascade of nonlinear light-tissue interactions such as photochemical processes, plasma formation, tissue expansion and protein denaturation due to high temperature occur^16^,^17^. Although the exact underlying principle for all these processes is not yet fully understood, it has been demonstrated that using a femtosecond (fs) pulsed laser for surgery allows for cutting of submicron-sized structures^18^,^19^,^20^ with minimal or negligible damage to surrounding tissue. This technique has been applied to the mammalian central nervous system, *in vivo* to produce vascular disruptions in rat brain parenchyma to model stroke^21^ and to dissect dendrites and ablate single spines in the rat cortex^22^, and *in vitro* to sever axons, which have submicron diameters^23^,^24^.

With such precision and flexibility in controlling dissection sites, we sought to cut the dendritic trees of pyramidal neurons and characterize the electrophysiological properties of neurons during and following dendrotomy. We monitor neurons electrophysiologically post dendrotomy and the separation is verified by evoking two-photon (2P) glutamate uncaging responses at identified spines proximal and distal to the site of dendrotomy and by biocytin staining *post hoc*. The effects of small- and large-extent dendrotomy on neuronal firing characteristics are investigated.

## Results

We used our previously reported custom-built 2P laser scanning microscope^25^,^26^ to obtain a 3D view of layer V (L5) cortical pyramidal cells (PCs) labelled with Alexa-488 from which we chose the site for dendrotomy. We used two separate lasers, one for imaging (at 800 nm) and another for dendrotomy (at 800 or 720 nm) and uncaging (at 720 nm). Our custom software allowed us to switch easily between the two lasers, modulate their intensities and position the laser beam at any arbitrary point for dendrotomy. We used a point excitation for dendrotomy of thin 3^rd^ and 4^th^ order dendrites and a line scan for thicker 1^st^ and 2^nd^ order dendrites.

**Figure 1:**
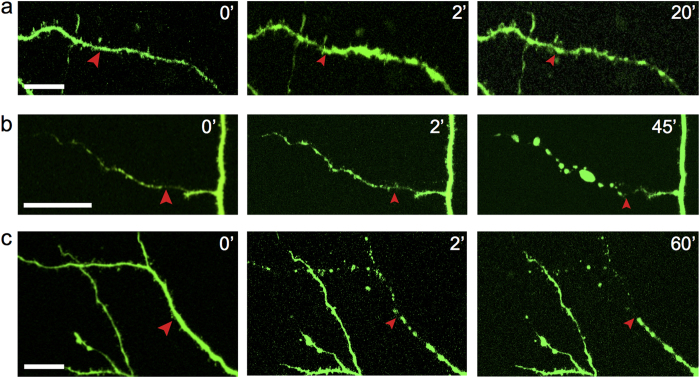
Morphological changes after dendrotomy. 2P fluorescence images showing dendritic segment before (0′) and after (2 min: 2′, 20 min: 20′) dendrotomy. (**a**) Swelling in distal dendritic segment, then recovery. (**b**) Sustained swelling during the observation period. (**c**) Beading, degeneration and disappearance of distal dendritic end. Arrows mark laser irradiation sites. Scale bars 20 μm.

Time series of 2P images were taken of the target site up to two hours after dendrotomy to examine the dendritic structure around the cut. We found that irradiating dendrites with laser light at power levels below a damage threshold induced no observable changes in morphology. Beyond the damage threshold, irradiation resulted in either swelling with recovery or complete transection (Fig. 1), consistent with previous reports^27^. As the power threshold for damage could not be determined *a priori*, the average power was increased in small increments starting from ~10 mW.

**Figure 2:**
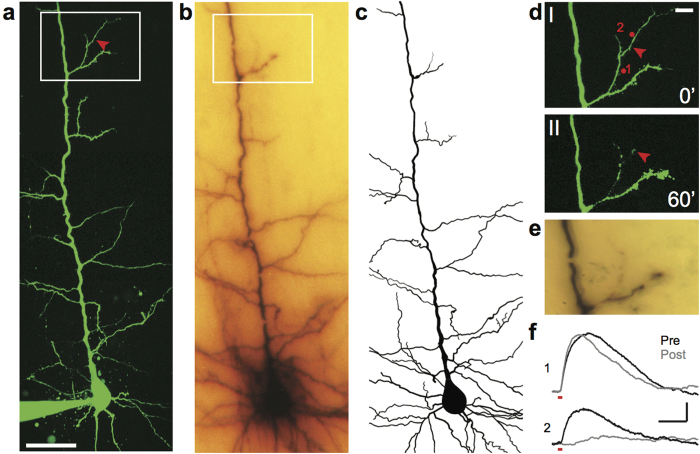
Histological and functional verification of dendrotomy in a 4^th^-order dendrite of a L5 pyramidal cell. (**a**) Montage of 2P fluorescence images of a L5 pyramidal cell prior to dendrotomy. Boxed region shows dendrotomy site. Red arrow indicates site of point scan for dendrotomy. Scale bar 50 μm. (**b**) Bright-field image of biocytin stain after histology with the same boxed region in *a* indicated. Breaks in the image are artefacts from embedding. (**c**) Neuronal reconstruction based on *b*. The axon and its collaterals were not reconstructed. (**d**) Magnified view of boxed area in *a* showing dendritic segment before (0′, I) and an hour after (60′, II) dendrotomy. Scale bar 10 μm. (**e**) Magnified view of boxed area in *b* showing histological evidence of dendrotomy. (**f**) EPSPs evoked by MNI-glutamate uncaging at spines proximal (1) and distal (2) to dendrotomy site prior to and post dendrotomy showing functional evidence of cut. Locations of sites are indicated in *d*I. Red lines under uncaging responses indicate uncaging laser pulse duration (4 ms). Scale bars 0.5 mV, 50 ms.

Figure 2a shows a L5 pyramidal cell where we performed dendrotomy in a 4^th^-order dendrite as indicated by the red arrow. To further assess structural integrity, slices were histologically processed *post hoc* and the relevant dendritic trees imaged (Fig. 2b) and reconstructed (Fig. 2c) using a bright-field microscope. Dendrotomy was considered successful if separation was evident in both the 2P (Fig. 2d) and bright-field images (Fig. 2e). To provide a functional assessment of dendrotomy, we performed localized 2P uncaging of MNI-glutamate at identified spines proximal and distal to the site of separation before and after dendrotomy. Successful dendrotomy was supported by the retaining of the amplitude of the proximal uncaging excitatory postsynaptic potential (uEPSP) but an insignificant distal uEPSP (Fig. 2f). This allowed us to rule out cases of incomplete dendrotomy.

**Figure 3:**
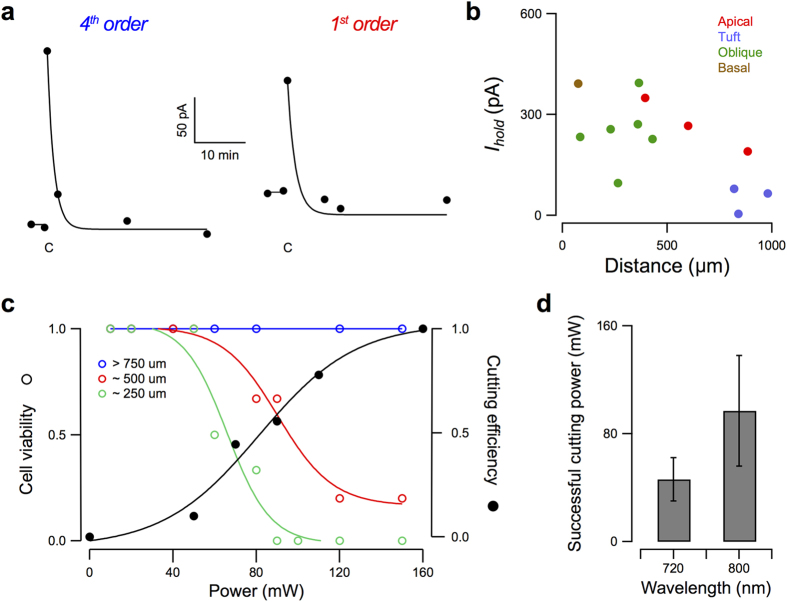
Electrophysiological monitoring of laser dendrotomy. (**a**) Representative time course plots showing sudden increase in *I*_*hold*_ with dendrotomy in 4^th^ order and 1^st^ order dendrites of L5 pyramidal cells at sites 600 μm and 360 μm away from the soma, respectively. *C* marks the time of laser irradiation. (**b**) Change in the magnitude of the neuron’s holding current, *I*_*hold*_, following dendrotomy for varying distances from the soma for different cells. (**c**) Dependence of cell viability and cutting efficiency on laser power. Cell is characterized as viable when *I*_*hold*_ returns to initial level or close to it after dendrotomy. The cut is considered successful when separation of the dendrite is observed in both the two-photon and biocytin images. Solid lines denote sigmoidal fit. (**d**) Average successful cutting power for two different wavelengths.

We monitored dendrotomy electrically through the holding current, *I*_*hold*_. We found that a sudden increase in the magnitude of *I*_*hold*_ was observable right after dendrotomy regardless of the location of the site along the dendritic tree (Fig. 3a). *I*_*hold*_ typically returned to close to initial values within ~10 minutes (10.6 ± 7.0 mW, *n* = 13), an indication that the dendrite may have resealed. The magnitude of the increase in *I*_*hold*_ decreased with increasing distance of the location from the soma (Fig. 3b).

We then investigated the effect of laser power on cell viability, defined as *I*_*hold*_ returning to the initial level or close to it ( ± 25 pA). Figure 3c compares cell viability with applied laser power for irradiation with 800 nm laser light at different distances from the soma. At regions close to the soma (∼250 μm), all cells (*n* = 3) remain viable with up to 50 mW applied laser power. Some are no longer viable with higher power levels until all become unviable with 90 mW applied power. Cell viability increases throughout the range of the laser power with laser irradiation farther from the soma (∼500 μm, *n* = 6). At distal regions (∼750 m from the soma), viability of the cells (*n* = 6) is maintained throughout the range of the laser power available. For comparison, the cutting efficiency is plotted against laser power. Expectedly, whereas cell viability generally decreases with laser power, the cutting efficiency increases. It may seem like the optimal power level for dendrotomy is the power where the cutting efficiency and cell viability plots intersect. For distances less than 750 μm from the soma, this occurs where the dendrotomy success rate is less than 60%. However, in our experiments we determined the optimal laser power for achieving successful dendrotomy while maintaining cell viability by systematically incrementing the laser power from a very low initial level until successful dendrotomy was observed. This allowed us to make successful cuts with the cells remaining viable in 91% (21/23) of experiments. Average power settings for dendrotomy were 97.0 ± 41 mW (*n* = 6) and 46.2 ± 16 mW (*n* = 7) using 720 and 800 nm laser light, respectively (Fig. 3d). As expected, a higher laser power is required for the lower-energy wavelength.

**Figure 4:**
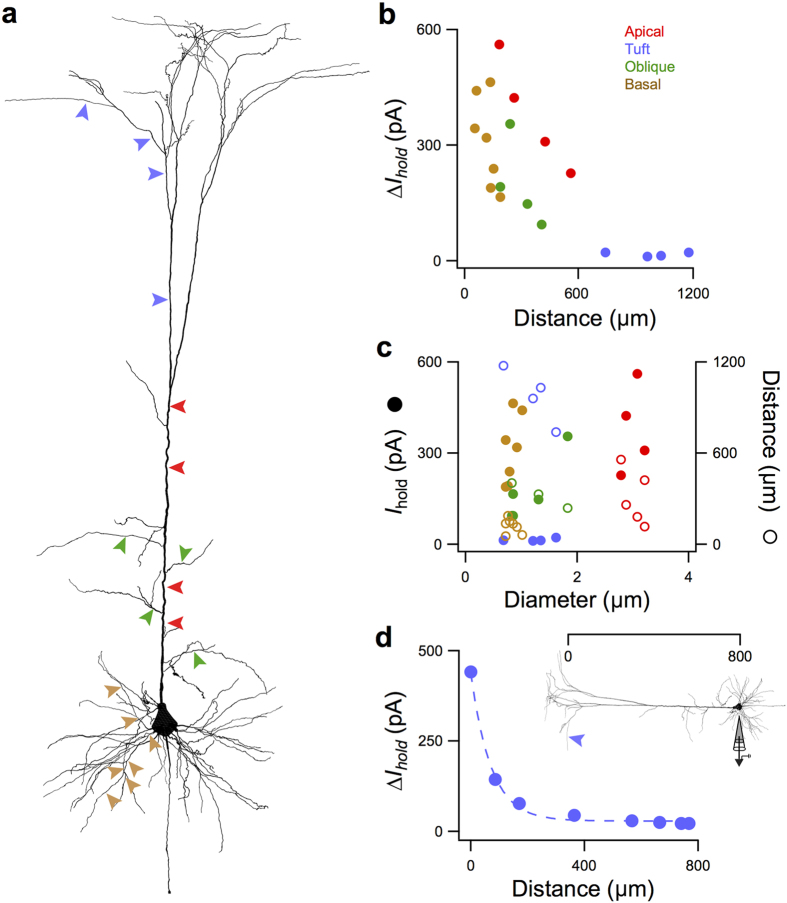
Numerical modelling of laser dendrotomy. (**a**) Schematic of L5 pyramidal cell used in the modelling. Dendrotomy sites are indicated with color-coding referring to apical (red), tuft (purple), oblique (green) and basal (brown) dendrites. (**b**) Change in the magnitude of the neuron’s holding current, *I*_*hold*_, following dendrotomy for varying distances from the soma. (**c**) Rise in *I*_*hold*_ (filled circles) and distance (open circles) from soma of dendrotomy site versus dendrite diameter. Change in *I*_*hold*_ increases with diameter except for the apical dendrite. Similarly, except for the main apical dendrite, dendrite diameter decreases with distance within each dendritic domain. (**d**) Rise in *I*_*hold*_ measured at different positions of the voltage-clamp electrode for dendrotomy at the site marked by purple arrow in the inset. Dashed line is an exponential fit.

We verified the observed rise in *I*_*hold*_ following dendrotomy with numerical simulations using the reconstructed morphology of one of the L5 pyramidal cells with active conductances (Fig. 4a). We simulated the open end of a cut dendrite by setting the membrane conductance (*g*_pas_) of the cut segment to 0.04 S/cm^2^ and its reversal potential (*e*_pas_) to 0 mV. Likewise, a sealed end was simulated by setting the axial resistance (*R*_a_) of the segment to 10^30^ Ωcm. We were able to reproduce the same inverse relationship between increase in *I*_*hold*_ and distance from the soma (Fig. 4b). This relationship was especially evident within the same dendritic domain (*i.e*. apical, oblique, basal dendrites, tufts) and is a consequence both of the dendrites within a domain decreasing in diameter with distance from the soma (Fig. 4c) and of current attenuation along the dendrite (Fig. 4d).

**Figure 5:**
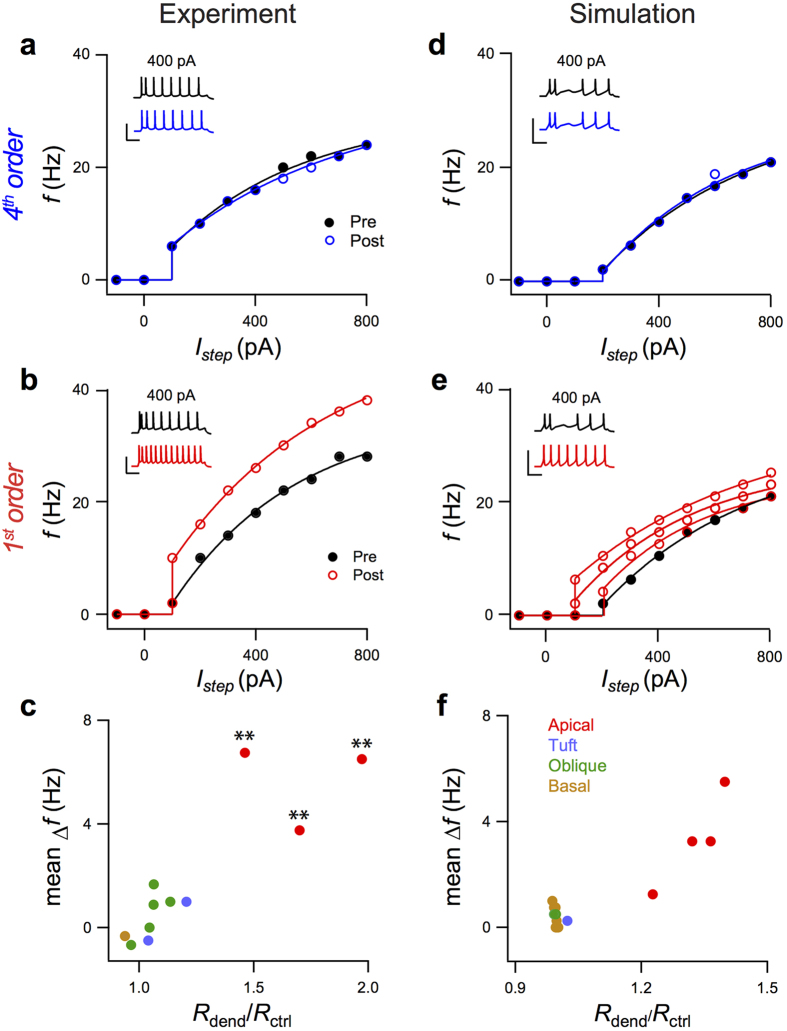
Changes in temporal firing pattern in response to current injection. Experimentally acquired *f-I* plots before (solid circles) and after (hollow circles) dendrotomy of representative (**a**) 4^th^ and (**b**) 1^st^ order dendrites, 600 μm and 360 μm from the soma, respectively. Solid lines denote exponential fit. The insets show representative firing patterns for *I*_*ste*p_ = 400 pA. Scale bars 80 mV, 100 ms. (**c**) Mean differences between pre- and post-dendrotomy firing frequencies acquired via experiment for different current step amplitudes plotted against the ratio of the value of *R*_*in*_ after dendrotomy to its initial value. **denotes *P* < 0.001 for comparison of corresponding *f-I* curves before and after dendrotomy. (**d**–**f**) Simulation plots similar to (**a**–**c**) Experimental data points in ***c*** correspond to different cells while in ***d***, all numerically acquired data points are from a single model cell. The dendrotomy sites are shown in Fig. 4a.

To characterize the neuron electrophysiologically before and after dendrotomy, we recorded the firing rate before dendrotomy and after *I*_*hold*_ returned close to the initial level. Figures 5a,b compare the firing rates before (solid circles) and after (open circles) dendrotomy for representative cuts of a 4^th^- and a 1^st^-order dendrite, 600 μm and 360 μm from the soma of L5 pyramidal cells, respectively. Dendrotomy of the 4^th^-order dendrite did not significantly change the frequency versus current (*f-I*) relationship (*P* > 0.2; Fig. 5a). It also did not alter the input resistances (80 ± 18 before *vs* 85 ± 7 MΩ after dendrotomy). However, dendrotomy of the 1^st^-order dendrite significantly increased the firing rate (*P* < 0.001; Fig. 5b) with a concomitant increase in input resistance (82 ± 23 vs. 141 ± 22 MΩ, respectively). Such an increase is consistent with the removal of a large area of dendritic membrane.

We investigated how the cutting of varying extents of dendritic membrane affected the *f*-*I* relationship. Figure 5c summarizes the change in firing frequency for dendrotomy at different locations in the dendritic tree corresponding to removal of different amounts of dendritic membrane. The difference between firing frequencies before and after dendrotomy was quantified as the mean difference over the different stimulus current amplitudes. The figure shows that dendrotomy of 2^nd^, 3^rd^ and 4^th^ order dendrites produces less change in dendritic membrane area (normalised change in *R*_in_ = 1.1 ± 0.1, *n* = 8) compared to dendrotomy of 1^st^-order dendrites (1.7 ± 0.3, *n* = 3) and consequently has a small influence on the firing rate. *f-I* curves were only significantly changed by dendrotomy of 1^st^-order dendrites (*P* < 0.001).

Using the same model neuron shown in Fig. 4a, we numerically reproduced the shift to higher frequencies as observed experimentally for 1^st^-order dendrites (Fig. 5d), but which was not present for 4^th^-order dendrites (Fig. 5e). Moreover, the plot of the difference between firing frequencies before and after dendrotomy versus the normalised input resistance (Fig. 5f) was qualitatively similar to the experimental observations, indicating that only a considerable removal of dendritic membrane has a significant impact on the *f-I* relationship.

## Discussion

We have demonstrated femtosecond laser dendrotomy which allows for highly localised dissection of individual dendritic branches. Dendrotomy was characterised by a sudden and large increase in *I*_*hold*_, which typically returned close to the initial level, suggesting resealing of the cut end.

Dendrotomy in 2^nd^-, 3^rd^-, and 4^th^-order dendrites did not change neuronal firing rate significantly. However, dendrotomy in 1^st^-order dendrites resulted in an increase in firing rate for the same current injected. The dendritic tree serves as an electrical load for action potential generation and removal of a large part of it lowers the electrical load on the neuron, consequently enhancing its excitability. Similar observations have been reported after dissection of the main apical trunk of Purkinje cells and L5 pyramidal cells^15^.

The morphological effect of laser irradiation was either beading or complete dissection. For cases where separation was not evident but beading in the dendrite persisted for the whole duration of observation, it is likely that the distal dendrite would have eventually degenerated and disappeared given a longer observation period. In dendrotomy performed *in vivo*, Sacconi *et al.*^22^ observed swelling within the first 40 minutes after dendrotomy but no dissection was evident until after 24 hours.

Here, success of dendrotomy was verified in three ways: 2P fluorescence imaging, glutamate uncaging and biocytin staining. In the case of 2P fluorescence imaging, it is important to note that structural integrity is inferred from the dye concentration, which in turn is inferred from the measured intensity. Loss of fluorescence, however, does not always equate to removal of material. Fluorescent dyes are susceptible to photobleaching. Bourgeois and Ben-Yakar^17^ observed the photobleaching effect in *C. elegans* axons right after laser irradiation. They observed that below the threshold causing damage, axons may lose fluorescence in irradiated regions despite incomplete axotomy. Thus, when concluding success of dendrotomy solely from the 2P fluorescence image, it is important to confirm the dissection again after 15 min.

The underlying working mechanism for femtosecond laser nanosurgery is not yet fully understood. In a modelling work that simulated plasma formation in water, the evolution of temperature distribution, thermoelastic stress generation and stress-induced bubble formation, Vogel *et al.*^16^ reported that nanosurgery with 80-MHz repetition rate lasers, such as is used here, is mediated by free-electron-induced chemical decomposition or bond breaking in conjunction with multiphoton-induced chemistry. It has been interpreted that such chemical decomposition of membrane components changes membrane permeability, leading to dendritic swelling^27^. If the effect is small enough, the neuron recovers. Otherwise, beadlike structures form and the distal dendritic end degenerates similar to how severed axons go into Wallerian degeneration^28^. With increased laser energy levels, damage may also be created by heating and long-lasting bubbles produced by tissue dissociating into volatile components^16^.

It is possible that the sudden increase in *I*_hold_ observed here is due to the recruitment of mechanosensitive (MS) channels^29^,^30^ that respond to mechanical perturbation of the membrane. Such perturbation may result from thermal molecular agitation or cell swelling^31^. Several MS channels belong to the transient receptor potential (TRP) superfamily^32^,^33^ and are present in the mammalian nervous system^30^. The role of MS channels may be tested in pharmacology experiments where dendrotomy is carried out with MS channel blockers.

Dendrotomy with minimal focal damage can only be achieved with an optical technique using a cascade of non-linear multi-photon processes. Non-optical techniques generate extensive collateral damage and are mostly restricted to transection of the main apical dendrite^11^,^12^,^13^,^14^,^15^. Here, we have successfully manipulated the dendritic topology by femtosecond laser dendrotomy. The capacity of this technique to dissect individual dendrites while leaving the rest of the dendritic tree intact enables *in vitro* studies of signal integration with little impact on the remainder of the tree and surrounding neuropile. For example, the interaction between currents from different cellular compartments and their contributions to synaptic integration^15^,^34^ could be studied. This technique could also be applied *in vivo*. Because alterations in dendritic topology have been observed in clinically diagnosed mental dysfunction such as Alzheimer’s^35^, epilepsy^36^, mental retardation^37^ and even prolonged stress^38^,^39^,^40^, an efficient experimental protocol to alter dendritic topology could pave the way for alternative approaches to studying these diseases.

## Methods

### Slice preparation and electrophysiology

All experiments were performed in accordance with the approved guidelines set by the Animal Ethics Committee of the Australian National University. Young male Wistar rats (P15-19) were rapidly decapitated and 300 μm thick parasagittal cortical brain slices were prepared using standard methods^25^.

For recordings, slices were perfused with carbogen-bubbled artificial cerebrospinal fluid (ACSF) at 34–35 °C. We filled L5 pyramidal cells through the recording patch pipette (*R* = 3.5–4.5 MΩ) with standard intracellular solution^25^ to which 0.1 mM Alexa-488 (Invitrogen) and 13.5 mM biocytin were added. Data were acquired with a MultiClamp 700B amplifier (Molecular Devices). Where necessary, the resting membrane potential of −65 mV was maintained by current injection. To evoke action potentials, current steps of 500 ms duration from −100 to 800 pA in increments of 100 pA were delivered at 1 Hz.

### Optical setup

We used a custom-built two-photon microscope as previously reported^26^. Two NIR laser beams, one for imaging (800 nm; MIRA 900, Coherent Scientific, Santa Clara, CA, USA) and another for uncaging (720 nm) and cutting (720 nm or 800 nm; Chameleon, Coherent Scientific), are combined ahead of the objective lens (40x 1.0 NA, Carl Zeiss, Jena, Germany) via a polarizing beam splitter following reorientation of the polarization of each laser using half-wave plates. Galvanometer scanning mirrors scan the linearly polarized beam for 2P fluorescence imaging and choice of location for uncaging or dendrotomy. A dichroic mirror reflects the beam to the objective. We obtain a 2P image by collecting the green fluorescence from the sample directed to a second dichroic mirror, which reflects wavelengths below 650 nm into a photomultiplier tube. The sample may also be viewed via an upright differential interference contrast (DIC) microscope (BX50WI, Olympus, Tokyo, Japan). In DIC imaging mode, the dichroic mirror above the objective lens allows infrared light (>810 nm wavelength) to pass through and focus onto a CCD camera (IR-1000EX, Dage-MTI, Michigan City, USA). Custom software developed in Labview (R2010a, National Instruments, Austin, Texas, USA) controls the acquisition of 2P fluorescence images and enables easy switching between the two lasers, the modulation of their intensities by driving the motorised mount of a half-wave plate, and the positioning of the laser beam at any arbitrary point for dendrotomy or uncaging.

### Two-photon fluorescence imaging, dendrotomy and glutamate uncaging

Neurons were filled for 20–30 min with 0.1 mM Alexa-488 and imaged at 800 nm with 12–22 mW laser power. Image stacks of 800 × 800 pixels in a single plane were generated by imaging individual planes in 1 μm increments along the *z*-axis. ImageJ (National Institutes of Health, USA) was used for 3D visualization.

The location for subsequent dendrotomy was chosen from the 3D fluorescence stack. 1^st^, 2^nd^, 3^rd^ and 4^th^ order dendritic segments were cut with 100 ms pulses at 800 nm. Dendrotomy of thin 3^rd^ and 4^th^ order dendritic segments was performed with a point excitation (spot size of 184 nm for λ = 800 nm) while dendrotomy of thicker 1^st^ and 2^nd^ order dendritic segments was done with a line scan (scanning frequency 100 Hz, 10 repetitions). The holding current of the neuron was monitored in whole-cell voltage clamp. We monitored the firing rate in current clamp before and after dendrotomy. Only experiments during which the holding current returned close to the initial value following the dendrotomy were incorporated in the analysis.

To functionally verify if dendrotomy was successful, EPSPs were generated via 2P uncaging of MNI-glutamate at identified spines proximal and distal to the locus before and after dendrotomy. The uncaging sites were determined from the 3D image prior to dendrotomy. MNI-caged glutamate (10 mM; Tocris Bioscience, Sapphire Bioscience, Waterloo, NSW, Australia) was locally delivered from a large-diameter pipette (1–2 MΩ) with a small pressure (**~**3 kPa) maintained from a syringe and uncaged at 720 nm. The laser pulse duration (2–4 ms) was controlled via a Uniblitz VS25 shutter (Vincent Associates, Rochester, USA). An automated drift correction algorithm was run before every uncaging trial to ensure that the spines remained optimally aligned with the beam.

Two separate lasers were used, one for imaging (at 800 nm) and another for dendrotomy (at 800 nm or 720 nm) and uncaging (at 720 nm). Laser power was set to 12–22 mW for imaging, 22–108 mW for dendrotomy and 9–30 mW for uncaging. As there was no way of predicting the power threshold for damage *a priori*, the power was increased in small increments starting from a low level, typically 10 mW. Average power settings for dendrotomy were 97.0 ± 41 mW and 46.2 ± 16 mW using 720 and 800 nm laser light, respectively.

### Histology and 3D reconstruction

After each successful recording, the pipette was slowly retracted from the cell, and the slice fixed in a 0.1 M phosphate buffer solution containing 4% paraformaldehyde and stored in the refrigerator. Histology was done based on previously published methods^41^,^42^. Stained neurons were imaged on a standard microscope (AxioSkop 2MOT, Zeiss, Germany) equipped with a high-resolution color camera. Neurons were reconstructed using Neurolucida software (version 10; MBF Bioscience, Williston, USA) and exported to NEURON (ver 7.3) simulating environment^43^ for visualization.

### Data analysis

Input resistances were calculated from subthreshold voltage deflections to intracellular current steps. The firing frequency, *f*, was plotted against stimulus current amplitude, *I*, and plots were compared using an F test. For simplicity in calculation, *f-I* curves were fitted with an exponential fit, which gave a significantly better fit than a line (typical exponential fit *p* = 0.4166; line, 0.0064).

### Neuronal modelling

Modelling was done using NEURON based on the 3D reconstruction of one of the L5 neocortical pyramidal cells that had been subjected to a 4^th^-order dendrotomy. The reconstruction was sub-divided into 1001 compartments. The distribution of passive properties and ion channels were taken from several models^44^,^45^ with *R*_*a*_ set 100 Ωcm, while *C*_*m*_ was set to 2.33 μF/cm^2^ for the axon, basals and soma and to 1.0 μF/cm^2^ for the other compartments. *R*_*m*_ was set to 11594 Ωcm^2^ for the axon, basals and soma and to 1.0 Ωcm^2^ for the other compartments. A hyperpolarization-activated cation channel (HCN), transient sodium channel (Nat), fast potassium channel (Kfast), slow potassium channel (Kslow), persistent sodium channel (Nap), muscarinic potassium channel (Km), slow calcium channel (Cas), calcium dependent potassium channel (KCa), and a calcium pump (CP) were inserted into different parts of the neuron with parameters described in Bahl *et al.*^45^. An open end of a cut dendrite was modelled by setting the membrane conductance (*g*_pas_) of the cut segment to 0.04 S/cm^2^ and its reversal potential (*e*_pas_) to 0 mV. A closed end of a cut dendrite was modelled by setting the axial resistance (*R*_a_) of the segment to 10^30^ Ωcm.

## Additional Information

**How to cite this article**: Ann Go, M. *et al.* Targeted pruning of a neuron's dendritic tree via femtosecond laser dendrotomy. *Sci. Rep.* **6**, 19078; doi: 10.1038/srep19078 (2016).
